# Management of malignant pleural mesothelioma: a French multicenter retrospective study (GFPC 0802 study)

**DOI:** 10.1186/s12885-015-1881-x

**Published:** 2015-11-06

**Authors:** C. Raynaud, L. Greillier, J. Mazieres, I. Monnet, B. Mastroianni, G. Robinet, G. Fraboulet, A. Dixmier, H. Berard, R. Lamy, J. Letreut, H. Lena, G. Oliviero, S. Botta, A. Vergnenegre, I. Borget, C. Chouaid

**Affiliations:** 1 Service de Pneumologie, CH Argenteuil, Argenteuil, France; 2 Service d’oncologie thoracique, APHM, Marseille, Services de Pneumologie, Marseille, France; 3 CHU Toulouse, Toulouse, France; 4 Service de pneumologie, CHI Créteil, 40 avenue de verdun, 94010 Créteil, France; 5 CHU Lyon, Lyon, France; 6 CHU Brest, Brest, France; 7 CH Cergy Pontoise, Cergy Pontoise, France; 8 CH d’Orléans, Orléans, France; 9 HIA Toulon, Toulon, France; 10 CH Lorient, Lorient, France; 11 CH Aix en Provence, Aix en Provence, France; 12 CHU Rennes, Rennes, France; 13 CH Longjumeau, Longjumeau, France; 14 CHU Rouen, Rouen, France; 15 CHU Limoges, Limoges, France; 16 IGR, Villejuif, France

**Keywords:** Malignant pleural mesothelioma, Treatment, Chemotherapy

## Abstract

**Background:**

Malignant pleural mesothelioma (MPM) is a rare disease with poor prognosis in spite of significant improvement in survival, due to new chemotherapy regimens. We describe here patients’ profiles and management in daily practice in France.

**Methods:**

Observational retrospective study. Data were collected from medical files. All patients with histologically proven MPM diagnosed from January 2005 to December 2008 were included in the participating sites.

**Results:**

Four hundred and six patients were included in 37 sites: mean age 68.9 ± 9.8 years, male predominance (sex ratio 3.27), latency of the disease 45.7 years, epithelioïd type 83 %. Diagnosis was made using thoracoscopy in 80.8 % of patients. Radical surgery was performed in 6.2 % of cases. Chemotherapy was administered to 74.6 % of patients. First line regimens consisted mainly of platinum + pemetrexed (91 %) or pemetrexed alone (7 %). Objective response rate was 17.2 % and another 41.6 % of patients experienced disease stabilization. Half of these patients underwent second line chemotherapy (platinium + pemetrexed 31.6 %, pemetrexed alone 24.6 %), resulting in a 6 % response rate. Third-line chemotherapy (56 patients) yielded disease control in 5.4 % of cases.

**Conclusions:**

The management of MPM in France is usually in accordance with guidelines. Response rates are somewhat lower than those described in clinical trials.

## Background

Malignant pleural mesothelioma (MPM) is a rare aggressive tumor. Since it is mainly associated with asbestos exposure, its incidence varies among countries and population sub-groups, depending on the degree of exposure.

The time between exposure and diagnosis of MPM often exceeds several decades. It has been shown recently that the risk continues to rise until 45 years following exposure [1]. The incidence of MPM is continuously increasing in some contries as Australia and United Kingdom [2–4] but remains very stable for over 5 years in U.S. and Japan. In France, the epidemiological pattern is different since asbestos use has been strictly controlled as early as 1978 and definitely forbidden in 1997. New cases are still diagnosed due to the long latency of the disease, but apparently, the incidence peak has been reached in 2000–2005 for men [5]. However, in the meantime, the incidence is still increasing in women, in whom professional exposure is often missing. The number of MPM-related deaths is approximately 1100/year in men and 300/year in women in France [5] while the incidence ranges from less than 1/million for the general population to 50–100/million for at-risk sub-groups [5]. A national network has been created in 1998 (PNSM: Programme National de Surveillance des Mésothéliomes) for epidemiological observation, clinical research and organization of healthcare supply [6]. Apart from the well-known occupational exposure, environmental exposure to asbestos or other carcinogenic compounds remains to be explained while they seem to account for more and more cases [4]. No oncogenic driver has been identified and molecular pathways leading to MPM are also unclear, so that up to now, there is no evidence for using specific targeted therapies in the treatment of these tumors and most clinical trials yielded negative outcomes [7]. Extrapleural pneumonectomy preceded by neoadjuvant chemotherapy and followed by hemithorax irradiation has been almost abandoned in routine practice, since several clinical trials have demonstrated low feasibility, absence of survival benefits and unacceptable postoperative morbimortality [8, 9]. Treatment of advanced diseases relies primarily on chemotherapy with a combination of platinum and pemetrexed until the results of ongoing clinical trials provide information that may change our practice (association with bevacizumab,new targeted therapies or immunotherapy).

The objective of our study was to describe the patients’ profiles and the management of MPM in France, as regards diagnosis and treatment.

## Methods

In this observational national multicenter study, we retrospectively reviewed the medical files of patients diagnosed and treated in France. All the sites belonging to the GFPC (French Group of Onco-Pneumology) were invited to participate. Patients were included if they had a histologically proven MPM diagnosed between January 2005 and December 2008, and if they had been managed in the participating site. Patients who were treated for recurrent mesothelioma during this period were excluded if the diagnosis had been made before January 2005. The following information was recorded: patients’ demographics, smoking status and medical history, exposure to asbestos (assess by a specific questionnaire), symptoms, diagnostic procedures, type of treatment (surgery, radiation therapy and chemotherapy) and response to treatment, as evaluated by local investigators.

Descriptive statistics were used, namely means, standard deviations, medians and ranges for quantitative variables, counts and percentages for categorical variables. No statistic tests were performed. The date of last observation was December 31, 2008. The protocol was approved by the CHU Limoges ethics committee, on behalf of all participating centers, and the study complied with good clinical practice and the Helsinki Declaration. All participants signed informed consent.

## Results

We included 406 patients diagnosed and managed at 37 sites (203 cases in 16 teaching hospitals, 183 in 18 general hospitals, 14 in 2 cancer research centers and 6 in one military hospital). Their main characteristics are summarized in Table 1. Men accounted for 77 % of the cohort; most frequent comorbidities were high blood pressure (37.7 %) and cardiovascular diseases (17 %). A majority of patients had respiratory symptoms at diagnosis (93 %), mainly dyspnea and thoracic pain. A previous exposure to asbestos was identified in 64 % of cases and the mean time from exposure to diagnosis was 45.7 years.

**Table 1:** Main characteristics of patients (*n* = 406)

Age at diagnosis, years (mean + SD)	68.9 ± 9.8
Gender, male	76.6 %
Smoking status (*n* = 367)	
Never smoker, n (%)	152 (42.4 %)
Current smoker, n (%)	36 (9.8 %)
Past smoker, n (%)	179 (48.8 %)
Pack-years, mean ± SD	28 ± 17.1
Medical history, n (%)	
Cancer	40 (9.8 %)
Chronic obstructive pulmonary disease	17 (4.2 %)
Cardiovascular disorders	69 (17.0 %)
High blood pressure	153 (37.7 %)
Neurovascular disorders	15 (3.7 %)
Asbestosis	7 (1.1 %)
Diabetes mellitus	54 (13.0 %)
Symptomatic at diagnosis (*n* = 373), n (%)	
Yes	347 (93.0 %)
Dyspnea	248 (71.5 %)
Thoracic pain	154 (44.4 %)
Other	158 (45.5 %)
Exposure to asbestos (*n* = 406), n (%)	259 (63.8 %)
Type of exposure (*n* = 259)	
Professional	251 (96.9 %)
Environmental	8 (3.1 %)

Examinations performed for disease staging included thoracic CT scan (98 %), abdomino-pelvic CT scan (65 %), echocardiography (22 %), PET-scan (24 %) and magnetic resonance imaging (10 %). Diagnosis was based on surgical thoracoscopic/pleuroscopic biopsy in most cases (Table 2). The epithelioïd type was the most frequent (82.9 %) followed by sarcomatoid (10 %) and mixed or biphasic (7.1 %) types.

**Table 2:** Diagnostic procedures (*n* = 406)

Blind closed pleural needle biopsy with local anesthesia	16 (3.9 %)
Medical thoracoscopic biopsy	11 (2.7 %)
Surgical thoracoscopic biopsy	317 (78.1 %)
Biopsy by thoracotomy	45 (11.1 %)
CT-scan guided blind closed pleural needle biopsy	16 (4 %)
Unknown	1 (0.2 %)

Only 30 patients underwent curative surgery, namely extra-pleural pneumonectomy and pleurectomy in 25 and 5 cases respectively. In addition, pleurodesis was performed in 191 patients. Two hundred and sixty-eight patients had prophylactic drain site radiation therapy with a mean number of 5.2 ± 6.2 sessions and a mean number of 6 ± 2.2 Gy per session.

**Fig. 1:**
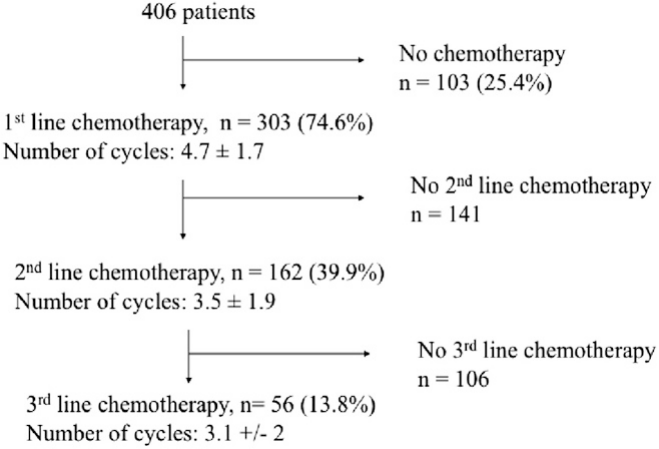
Chemotherapy administration

**Fig. 2:**
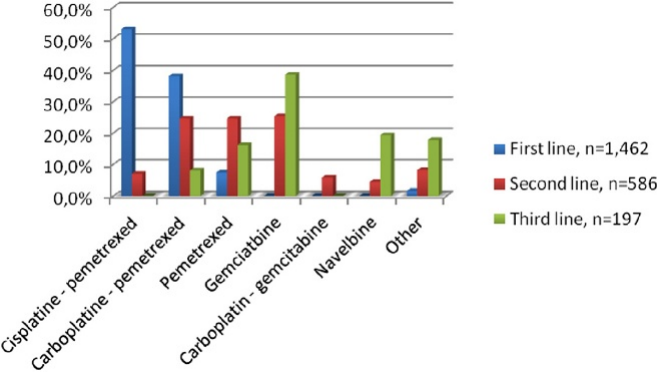
Chemotherapy regimens used in first, second and third line (% of cycles)

First-line chemotherapy was administered to 303 (74.6 %) patients, among whom 162 (53.5 %) and 56 (18.5 %) had second- and third-line chemotherapy respectively (Fig. 1). Most frequently used chemotherapy regimens were cisplatin – pemetrexed in first line, and gemcitabine as single agent in 2^nd^ and 3^rd^ line (Fig. 2).

The mean (median) numbers of cycles were 4.7 ± 1.7 [6], 3.5 ± 1.9 [3] and 3.1 ± 2 [3] in first, second and third lines respectively. The percentage of patients who responded to or were stabilized by treatment decreased over line, from 60.1 % in first line to 5.3 % in third line chemotherapy (Table 3).

**Table 3:** Clinical response

First line chemotherapy (*n* = 303)	
Complete response	9 (3.0 %)
Partial response	43 (14.2 %)
Stable disease	126 (41.6 %)
Disease control (CR + PR + SD)	178 (58.8 %)
Progression	99 (32.7 %)
Non evaluable	26 (8.5 %)
Second line chemotherapy (*n* = 162)	
Complete response	1 (0.6 %)
Partial response	9 (5.4 %)
Stable disease	31 (18.6 %)
Disease control (CR + PR + SD)	41 (24.6 %)
Progression	78 (46.7 %)
Non evaluable	48 (28.7 %)
Third line chemotherapy (*n* = 56)	
Complete response	0
Partial response	1 (1.8 %)
Stable disease	2 (3.6 %)
Disease control (CR + PR + SD)	3 (5.4 %)
Progression	27 (48.2 %)
Non evaluable	26 (46.4 %)

## Discussion

We described here patients’ characteristics and management of MPM in France. This patient cohort represent only 10 % of all patients diagnosed and treated in France; but it should be highlighted that all high volume centers participated and the remaining patients are treated individually throughout the country in centers with very low volumes. Since MPM is a rare disease, there are few such reports in the literature. A Turkish series of 228 patients diagnosed and followed from 1993 to 2010 was published in 2012 [10]. Age at diagnosis was 10 years younger in the Turkish cohort (59 years) and sex ratio was only 1.4 in favor of men. The type of exposure was probably different since most patients were farmers and housewives, and lived in rural areas. Diagnostic procedures were also different since blind closed pleural needle biopsy was performed in more than one half of patients and thoracoscopy in only 3 %. In another Turkish cohort of 54 patients from a single center, age was also younger (60.3 years for men), male predominance was moderate (55.6 %) and epithelioïd type accounted for only 50 % of the tumors [11]. These findings suggest that the epidemiology of mesothelioma is different in France and Eastern European countries, where tumors are more frequent in women and occur at younger ages, with a different distribution of histological types. Indeed, it is well known that in Turkey, environmental exposure is frequent due to opencast asbestos mines, and genetic predisposition has been shown in some regions [12].

The latency in our cohort was 45.7 years, which is consistent with other epidemiological studies [1, 3, 4]. It has been shown that apart from intensity and duration of exposure, the risk of MPM is inversely correlated with the age at first exposure [13]. However, this information has not been recorded in our patients’ files.

During the 2003–2007 5-year period, the pathological branch of the PNSM network (a mandatory, national review) analyzed 1348 pleural biopsies, among which 82 % were malignant mesotheliomas [6]. In this large sample of MPM, 80 % were epithelioïd, 6 % were sarcomatoid and 11 % were biphasic. Although our study included only 1/10^th^ of the estimated total number of cases of MPM diagnosed in France in the same period, the distribution of histological types is very similar to ours and these data suggest that our cohort is representative of MPM in France.

Main guidelines in the diagnosis and management of MPM are those issued by the European Respiratory Society - European Society of Thoracic Surgeons (ERS-ESTS) [14] and those issued by the European Society of Medical Oncology (ESMO) [15]. The use of positron emission tomography (PET) scanning is still under debate because MPM tends to only grow locally and metastases occur solely in patients with advanced disease but PET scanning can be used in the diagnostic work-up when

PET-avid sites in the thoracic cavity need to be identified to obtain representative tissue. In this analysis, only 24 % of the patients had a Pet scan as initial procedure; this can be explain by a limited access to this test in France, particularly at the study period. From a diagnostic perspective, thoracoscopy is recommended by ERS-ESTS for the pathological diagnosis of MPM, using deep and large biopsies while cytology and fine needle biopsies are considered insufficient. The ESMO also states that histology is the gold standard, using pleuroscopy or open pleural biopsy. In our series, almost all atients had a MPM diagnosis based on histology, biopsies being obtained by thoracoscopy and thoracotomy in the majority of cases.

Few options are available for the treatment of MPM and its prognosis remains very poor with a median overall survival below 12 months [16]. Extrapleural pneumonectomy does not offer survival benefits and it is accompanied by high morbidity and mortality [9, 17]. Both ERS-ESTS and ESMO guidelines recommend that extrapleural pleurectomy be performed only in a palliative setting to obtain symptoms control, and that radical surgery be performed only in highly specialized centers, as part of clinical trials, in a multimodal approach. In our series, only 30 (7.4 %) patients underwent surgery with curative intent. Of note, the results of the MARS study [9] had not been published at this time.

Prophylactic irradiation of tracks, intending to avoid tumor seeding after chest drain or pleural biopsy, is not recommended due to discrepancies between the few studies and to insufficient data [8–21]. Despite this recommendation, in our study, a large part of patients, 65.5 % received a prophylactic irradiation of tracks.

According to guidelines, when the decision is made to administer chemotherapy, first line treatment should consist of platinum associated with pemetrexed or raltitrexed [14, 22, 23]. This recommendation was followed by the French sites in our study since 91 % of first-line regimens consisted of platinum + pemetrexed.

In the initial study published by Vozelgang et al. in 2003, pemetrexed plus cisplatin was compared to cisplatin alone in chemonaïve patients [22]. The combination with pemetrexed resulted in longer overall survival (12.1 vs 9.3 months, *p* = 0.02), and increased response rates (41.3 vs 16.7 %, *p* < 0.0001). In our cohort, only 17.5 % of patients responded to this first-line chemotherapy, while the median number of cycles [6] was similar. According to the preliminary results of an Italian cohort of patients (*n* = 322) receiving pemetrexed alone (28 %) or associated with cisplatin or carboplatin, the overall response rate was 28.5 % [24]. However, in the phase II study published in 2006 by Ceresoli et al., only 18.6 % of patients treated with pemetrexed + carboplatin (19/102) had an objective response, including 2 CR and 17 PR, while 65.7 % achieved disease control, which is more consistent with our findings [25]. However, the response rate observed in our retrospective cohort seems low and has to be considered with caution. Indeed, in our study, there was no centralized review of the response to treatment and the follow-up of the patients was not standardized.

Drugs to be used in second line are not well-defined: guidelines suggest, with low levels of evidence, re challenge with the initial combination in case of response, or vinorelbine, or inclusion in clinical trials [14, 15]. In our series, the response rate was very low in the second-line setting (6 %) and only 24.6 % of patients had disease control. This result rate is consistent with that observed in a US study (2 %) that retrospectively reviewed the outcome of gemcitabine and/or vinorelbine chemotherapy in pemetrexed pretreated patients [26]. However, in this series of 60 patients, 46 % had stable disease, which is much higher than in ours. In another trial, vinorelbine single agent administered as second or further line chemotherapy in pemetrexed pretreated patients yielded a 15.2 % response rate and 33.9 % stable disease [27]. In a Japanese study, gemcitabine + vinorelbine administered as second line or beyond to pemetrexed pretreated patients resulted in 18 % response rate and 82 % disease control [28]. Retreatment with pemetrexed (alone or in combination, second or further line) appears a valuable option with a 19 % response rate and 48 % disease control rate in an Italian study [29]. Response to rechallenge appears to be correlated with response to first line pemetrexed-based chemotherapy.

Due to the methodology of our study, several data are missing because they are not systematically reported in medical records in routine practice. We could not establish survival curves since we did not collect longitudinal data. However, we could show that our patients are representative of the French MPM patients, that recommendations are appropriately followed with regards to both diagnostic procedures and treatment, and that unfortunately, treatment outcomes in daily practice are not as good as those reported in clinical trials. Actually, new treatment options are urgently needed to improve patients’ prognosis. Targeted therapies yielded disappointing results up to now [7] and further results are eagerly awaited, such as those of the MAPS study [30]. In the recent ASCO congress, promising results from new and original approaches such as arginine deprivation [31] or vaccines [32] were reported. However, these data must be considered very cautiously, until they are confirmed in further studies.

## Conclusion

We showed in this study that the management of MPM in France is in line with European recommendations. Yet, treatment outcomes remain disappointing, usually lower in routine practice than in clinical trials. New treatment options are urgently needed to improve the prognosis of MPM.
